# Treatment of Upper Limb Paresis With Repetitive Peripheral Nerve Sensory Stimulation and Motor Training: Study Protocol for a Randomized Controlled Trial

**DOI:** 10.3389/fneur.2020.00196

**Published:** 2020-03-25

**Authors:** Adriana B. Conforto, André G. Machado, Isabella Menezes, Nathalia H. V. Ribeiro, Rafael Luccas, Danielle S. Pires, Claudia da Costa Leite, Ela B. Plow, Leonardo G. Cohen

**Affiliations:** ^1^Departamento de Neurologia, Hospital das Clínicas, São Paulo University, São Paulo, Brazil; ^2^Hospital Israelita Albert Einstein, Instituto Israelita de Ensino e Pesquisa, São Paulo, Brazil; ^3^Núcleo de Apoio à Pesquisa em Neurociências (Center for Interdisciplinary Research on Applied Neurosciences: NAPNA), São Paulo University, São Paulo, Brazil; ^4^Departament of Neurosciences, Lerner Research Institute, Cleveland Clinic, Cleveland, OH, United States; ^5^Cleveland Clinic Lerner College of Medicine, Case Western Reserve University, Cleveland, OH, United States; ^6^LIM 44, Department of Radiology, Faculdade de Medicina, Hospital das Clínicas/São Paulo University, São Paulo, Brazil; ^7^Human Cortical Physiology and Stroke Neurorehabilitation Section, National Institute of Neurological Disorders and Stroke, National Institutes of Health, Bethesda, MD, United States

**Keywords:** stroke, repetitive peripheral nerve sensory stimulation, sensory stimulation, motor rehabilitation, upper limb

## Abstract

**Background:** Repetitive peripheral nerve sensory stimulation (RPSS) has emerged as a potential adjuvant strategy to motor training in stroke rehabilitation. The aim of this study is to test the hypothesis that 3 h sessions of active RPSS associated with functional electrical stimulation (FES) and task-specific training (TST) distributed three times a week, over 6 weeks, is more beneficial to improve upper limb motor function than sham RPSS in addition to FES and TST, in subjects with moderate to severe hand motor impairments in the chronic phase (>6 months) after stroke.

**Methods:** In this single-center, randomized, placebo controlled, parallel-group, double-blind study we compare the effects of 18 sessions of active and sham RPSS as add-on interventions to FES and task-specific training of the paretic upper limb, in 40 subjects in the chronic phase after ischemic or hemorrhagic stroke, with Fugl-Meyer upper limb scores ranging from 7 to 50 and able to voluntarily activate any active range of wrist extension. The primary outcome measure is the Wolf Motor Function Test (WMFT) after 6 weeks of treatment. The secondary outcomes are the WMFT at 3, 10, and 18 weeks after beginning of treatment, as well as the following outcomes measured at 3, 6, 10, and 18 weeks: Motor Activity Log; active range of motion of wrist extension and flexion; grasp and pinch strength in the paretic and non-paretic sides (the order of testing is randomized within and across subjects); Modified Ashworth Scale; Fugl-Meyer Assessment-Upper Limb in the paretic arm; Barthel Index; Stroke Impact Scale.

**Discussion:** This project represents a major step in developing a rehabilitation strategy with potential to have impact on the treatment of stroke patients with poor motor recovery in developing countries worldwide. The study preliminarily evaluates a straightforward, non-invasive, inexpensive intervention. If feasibility and preliminary efficacy are demonstrated, further investigations of the proposed intervention (underlying mechanisms/ effects in larger numbers of patients) should be performed.

**Trial Registration:** NCT02658578.

## Introduction

Upper limb paresis occurs in up to 80% of subjects with stroke. Six months after stroke, two-thirds of survivors are not able to perform activities of daily living using their paretic hand and often do not return to work (1, 2). The catastrophic burden of stroke and the paucity of evidence-based rehabilitation interventions to decrease upper limb disability prompted research about effects of somatosensory stimulation in the form of repetitive peripheral nerve sensory stimulation (RPSS) to enhance motor function.

In RPSS, trains of electric pulses are delivered to peripheral nerves by surface electrodes. Intensities of stimulation are adjusted in order to elicit paresthesias, but not pain or movements, in the territory of the stimulated nerve. The goal of this intervention is to provide controlled enhancement of sensory input from the stimulated body part. The paradigm of stimulation was based on reports of changes in the sensory and motor cortices of animals induced by specific patterns of sensory stimulation. Duration of pulses of 1 ms and the frequency of bursts (10 Hz, 500 ms on, 500 ms off) were chosen to optimally activate proprioceptive and large cutaneous sensory fibers (3).

In 2000, in the first publication about RPSS in humans, ulnar nerve stimulation led to increase excitability of the motor cortex to transcranial magnetic stimulation in healthy subjects (4). Other studies confirmed and extended these findings (5–7). The encouraging results in healthy subjects and the key role played by sensory input on motor recovery in subjects with stroke (8) fostered research about the effects of RPSS on motor control of patients with upper limb paresis. In 2002, preliminary results indicated that a single session of RPSS might enhance strength of the paretic hand in subjects with stroke (9). Since then, other studies reported benefits of single or repeated sessions of RPSS in stroke [for a systematic review, see Conforto et al., (10)]. The bulk of the work of RPSS in stroke had focused in patients with mild to moderate motor impairments until 2016, when improvements exceeding minimal clinically important differences in the Active Research Arm Test, were reported in patients with moderate to severe impairments when 2-h RPSS was followed by 4 h of intensive task-oriented motor training for ten consecutive weekdays (11).

Six hours of daily rehabilitation for 10 days over 2 weeks may not be a feasible strategy in developing countries where the greatest disability from stroke occurs (12). Here, we report the design of the study “Peripheral Nerve Stimulation and Motor Training to Enhance Hand Function After Stroke” (ClinicalTrials.gov identifier NCT02658578). The aim of this study is to test the hypothesis that 3-h sessions of active RPSS associated with functional electrical stimulation (FES) and task-specific training (TST) distributed three times a week, over 6 weeks, is more beneficial to improve upper limb motor function than sham RPSS in addition to FES and TST, in subjects with moderate to severe hand motor impairments in the chronic phase after stroke.

## Methods and Analysis

### Design

**Figure 1:**
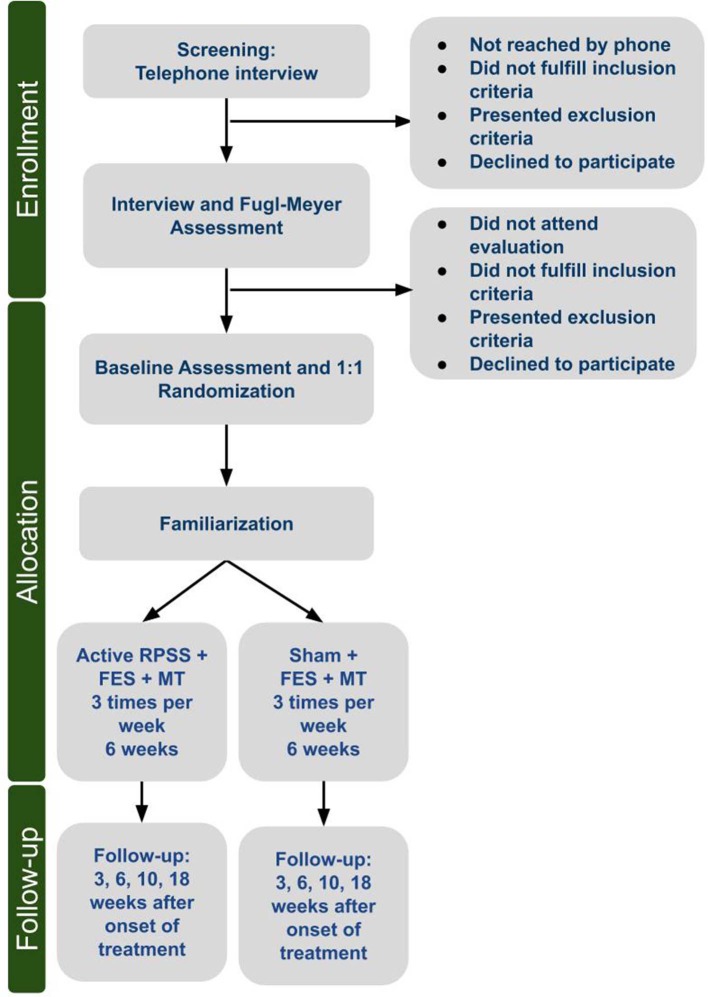
Flow chart.

In this single-center, randomized, placebo controlled, parallel-group study we compare the effects of 18 sessions of active and sham RPSS as add-on interventions to FES and task-specific training of the paretic upper limb, in subjects in the chronic phase after stroke. Patients are naïve to the hypotheses of the study. Patients and researchers responsible for evaluation of outcomes are blinded to treatment allocation. Figure 1 shows the flow diagram of the trial.

### Location and Setting

The study is being conducted at the Neurostimulation Laboratory at Hospital das Clínicas/São Paulo University in São Paulo, Brazil. Brazil is characterized by extreme social disparities and 4 in 5 inhabitants do not have private health insurance (13). Access to the public services is limited, in particular for specialized care such as neurological clinics and stroke rehabilitation. The majority of the population that receives care at Hospital das Clínicas/São Paulo University, the largest public, academic hospital in South America, is socially and economically disadvantaged. In the city of São Paulo, 1/3 of all subjects, and 1/2 of elderly subjects who attended university hospitals were found to be illiterate (14). In a study performed in our Emergency Department in São Paulo in 2003, 94.4% of the patients did not have private health insurance and 54.5% of the patients had >4years of education (15).

### Participants

#### Eligibility Criteria

Eligibility criteria are shown in Table 1. The ability to perform finger extension was not a prerequisite for inclusion.

**Table 1:** Eligibility criteria.

**INCLUSION CRITERIA**
18 years or older
Ischemic or hemorrhagic stroke at least 6 months before, confirmed by computed tomography or magnetic resonance imaging
Moderate to severe motor upper limb impairment defined as a score between 7 and 50 in the Fugl-Meyer Assessment of sensorimotor recovery after stroke (0–66) (15)
Ability to provide written informed consent (patient or legal representative)
Ability to comply with the schedule of interventions and evaluations in the protocol
**EXCLUSION CRITERIA**
Lack of ability to voluntarily activate any active range of wrist extension
Anesthesia of the paretic hand
Lesions that affect the cerebellum or cerebellar/vestibular pathways in the brainstem
Severe spasticity at the paretic elbow, wrist, or fingers, defined as a score of >3 on the Modified Ashworth Spasticity Scale
Active joint deformity
Uncontrolled medical problems such as end-stage cancer or renal disease
Pregnancy
Seizures, if current use of drugs that may decrease seizure threshold such as tryciclic antidepressants
Pacemakers
Other neurological disorders such as Parkinson's disease
Psychiatric illness including severe depression
Aphasia or serious cognitive deficits that preclude comprehension of the experimental protocol or ability to provide consent
Treatment of upper limb spasticity with botulinum toxin within the past 3 months

We expect to include patients with low levels of education, reflecting the characteristics of our population, shared with those of other low- and middle- income countries.

We include patients with ischemic or hemorrhagic stroke because even though mortality in the acute phase is greater in hemorrhagic than in ischemic strokes, survivors of hemorrhagic strokes and ischemic strokes with comparable impairments have similar responses to rehabilitation interventions (16, 17).

We do not include patients with hand anesthesia because there is evidence that RPSS exerts its effects by enhancing excitability in the sensorimotor cortex (4, 5, 7, 18, 19) and therefore, residual sensory function and partial integrity of cortical motor neurons are likely to be necessary for the beneficial effects of RPSS.

Children will not be included in the study because the question addressed by the proposal involves adult stroke patients. Stroke is rare in children, and the epidemiological profile, treatment, and prognosis of stroke is different in children than adults. Activities of daily living are also different in different developmental stages. The interventions in the current proposal are based on activities commonly performed by adults in daily living. Therefore, inclusion of children is inappropriate in the current proposal. A separate, age-specific study in children is warranted and preferable.

We exclude pregnant women because safety of RPSS has not been determined in this group. The study does not include other vulnerable populations such as prisoners or institutionalized individuals.

We exclude patients with other neurological disorders and psychiatric illness to prevent bias in the investigation of the experimental hypotheses. For the same reason, patients and caregivers are blinded to the experimental hypotheses.

#### Recruitment

Subjects are recruited in the community through advertisements on websites or local radio stations, and also among patients from the Stroke group/Neurology Clinical Division, Hospital das Clinicas/São Paulo University.

#### Subjects' Characteristics

The following characteristics are evaluated at baseline: age, gender, ethnicity, years of education, medications, time from stroke, type of stroke (ischemic/hemorrhagic), lesion side/location, scores in the National Institutes of Health Stroke Scale (NIHSS), (20) Modified Rankin Scale, (20) Minimental State Examination, (21) handedness prior to the stroke according to the Oldfield Inventory, (22) spasticity in elbow, wrist and finger joints in the paretic upper limb according to the Modified Ashworth Scale (MAS), (23) scores in the Fugl-Meyer Assessment of Sensorimotor recovery (upper limb—FMA-UL), (24, 25) Barthel Index (BI), (20) Stroke Impact Scale, (26) Beck Depression Inventory Short Form, (27) Self Reporting Questionnaire, (28) and DSM-V criteria for major depression (29). For ischemic strokes, etiologies are defined according to TOAST criteria (30).

In order to characterize brain lesions, magnetic resonance imaging (MRI) is performed in all patients before the first session of treatment, except in the case of contraindications, on a 3 T Phillips scanner, and include high-resolution 3D T1-weighted structural images (resolution = 1 mm^3^, matrix size = 240 × 240 mm^2^, field of view = 240 × 240 mm^2^, TR = 7 ms, TE = 3.2 ms, TI = 900 ms, flip angle = 8 degrees) and axial fluid-attenuated inversion recovery (FLAIR) and T2 images (4.5 inclx 4.5 × 4.5 mm^3^). In addition, diffusion sensitized gradients (voxel size = 2 mm, isotropic; TR = 10200 ms; TE = 103 ms) are applied along 64 collinear directions with a b value = 1000 s/mm^2^. In addition, two diffusion-weighted B0 images are obtained.

Lesion locations are defined by an experienced radiologist by evaluation of FLAIR, T2 and T1-weighted images as: right/left; frontal/temporal/occipital/parietal/insular; corticosubcortical, cortical or subcortical; with or without involvement of the precentral gyrus, postcentral gyrus, centrum semiovale, corpus callosum, posterior limb of the internal capsule, thalamus, basal ganglia, mesencephalon, pons or medulla.

In order to characterize the severity of motor involvement from a neurophysiological perspective, the presence or absence of motor evoked potentials to transcranial magnetic stimulation (TMS) is also registered at baseline, in the absence of contraindications (31). A safety screening questionnaire is filled before the procedure, as previously described (32). TMS is delivered to the affected hemisphere at paretic abductor pollicis brevis “hot spot” through a figure-of-eight shaped coil (Double 70 mm coil, maximum dB/dT, 25 kT/s) held by an investigator, connected to a Magstim 2002 monophasic stimulator (MagStim, UK). EMG activity was recorded at rest from surface electrodes placed over the affected APB. EMG responses are amplified (x1000), filtered (2 Hz−2 kHz) and sampled at 5 kHz with a computerized data acquisition system built with the LabVIEW graphical programming language (33). The intervals between TMS pulses are randomized between 5 and 7 s. If no motor evoked potentials can be elicited at 100% of the stimulator's output, TMS is then delivered through a MCF-65 figure-of-eight shaped coil [outer diameter, 75 mm; maximum initial dB/dT, (33) kT/s near the coil surface] connected to a MagPro X Compact (MagVenture). If no MEPs are registered at 100% of the stimulator's output, they are registered as “absent.”

### Randomization, Allocation Concealment and Blinding

Subjects are randomized by the Principal Investigator (PI) with a basic random number computerized generator (randomization.com) in blocks of four, to active or sham RPSS in a 1:1 ratio. The randomization schedule is concealed in a locked cabinet accessed only by the PI and the investigators who administer RPSS.

Participants and investigators who assess outcomes are blinded to the interventions performed. Subjects are blinded to the experimental hypothesis and are not allowed to discuss their experience during the interventions with the researchers involved in assessment of outcomes or with other subjects. The clinical research forms of the blinded intervention are kept concealed in a locked cabinet. After each intervention session, subjects are asked: do you think your nerves were stimulated (yes/no)? Do you think your hand is different (worse/better/no change)?

### Interventions

**Figure 2:**
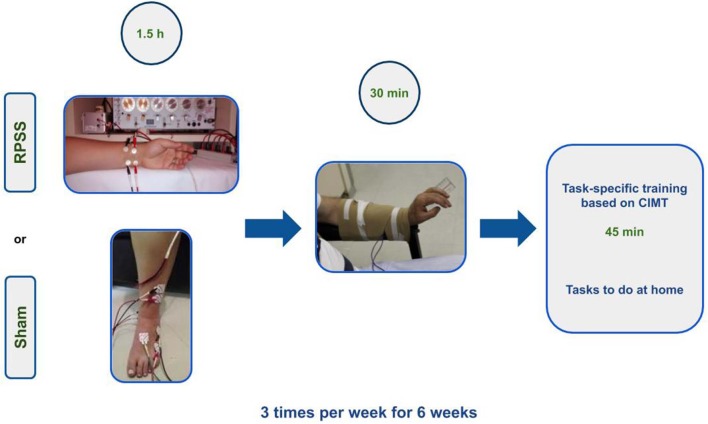
Experimental paradigm.

Figure 2 summarizes the experimental paradigm. Trained physical therapists administer interventions and assessed outcomes. Compliance with the interventions is monitored by one of the researchers. If a subject misses a session, the researcher calls him/her or the caregiver, in order to ascertain reasons for the absence and reinforce the importance of adherence to the protocol. All interventions are administered in the morning and scheduled according to the best possible time to subjects and caregivers. Transportation costs are covered by the protocol. The Template for Intervention Description and Replication (TIDieR) Checklist is shown in Supplementary Table 1.

#### RPSS

Trains of electrical pulses (1 ms of duration for each) at a frequency of 10 Hz are delivered at 1 Hz (500 ms on, 500 ms off) by three pairs of surface electrodes (KendallTM) with a square pulse stimulator (Grass Instrument Division of Astro-med, Inc, Braintree, MA) (34, 35). A customized device (Alfamedic Ltda, Sao Paulo) provides independent outputs to the three nerves, with a maximum output voltage of 130 volts.

In the active group, RPSS is administered to the median, ulnar and radial nerves of the paretic arm in all sessions. In the sham group, RPSS is administered to the superficial peroneal, tibial, and sural nerves of the paretic leg, as previously reported (34, 35). In both groups, the duration of stimulation is 90 min.

The threshold and maximum intensity of stimulation to produce paresthesias in the territory of the stimulated nerves, in the absence of visible muscle contractions or pain are determined. The intensity is kept as the maximum that evokes paresthesias in the absence of finger movements or pain (5, 9, 35).

Subjects are comfortably seated and are kept at rest during RPSS. Every 5 min, they are asked about the sensations elicited by stimulation in order to raise attention to the stimulated body part. The intensity of stimulation is increased if intensity of paresthesias decreases, and is decreased if movements or pain are elicited during the 90-min period of RPSS. During RPSS, they watch a movie to avoid fluctuations in wakefulness/attention (36).

In active RPSS, the median nerve is stimulated between the tendons of the flexor carpi radialis and palmaris longus with the cathode positioned 2–3 cm proximal to the wrist. The ulnar nerve is stimulated radially to the flexor carpi ulnaris tendon, with the cathode 3–5 cm proximal to the wrist. The radial nerve is stimulated 4–6 cm proximally to the ulnar styloid process (37). In all subjects, the three nerves are stimulated.

In sham RPSS, the superficial peroneal nerve is stimulated medially and about 12 cm proximally to the lateral malleolus. The tibial nerve is stimulated posteriorly and proximally to the medial malleolus. The sural nerve is stimulated in the midcalf, about 10–14 cm proximally to the lateral malleolus. This strategy for sham stimulation has been previously described (34, 38).

Researchers involved in RPSS receive training prior to participation in the protocol. They do not participate in FES, motor training or evaluation of outcomes.

#### Functional Electrical Stimulation (FES)

Immediately after active or sham RPSS, FES is delivered by a trained occupational or physical therapist to the extensor digitorum communis muscle of the paretic arm with self-adhesive (3 × 5 cm) electrodes and a FESMED IIV stimulator (CARCI, São Paulo, Brazil) for 25 min, after 5 min of familiarization (32). Square biphasic pulses with duration of 250 μs with auditory pace (human voice) are delivered at 0.4 Hz. Subjects are comfortably seated with their trunk and upper limb stabilized with bands. They are instructed to extend the wrist every time they hear the sound of a human voice saying “now” and then relax. A goniometer is kept next to the wrist to maintain the reference of target range of the movement, defined by, at least, 10 degrees of wrist extension.

#### Motor Training

Task-specific training based on Constraint-induced Movement Therapy (CIMT) (39) is applied to the paretic arm for 45 min. Four shaping tasks are defined for each patient and two tasks are applied per day. The tasks are chosen by a trained therapist based on the motor potential for each patient. Motor training is provided in an individual basis to each subject.

Two behavioral strategies are applied in each session of treatment. First, participants are asked about performance of 15 items of the Quality of Movement Scale of the Motor Activity Log (MAL) (24) to reinforce the use and perception of the upper limb in daily life. Second, subjects are assigned 10 functional tasks to be performed daily at home with the paretic arm, for at least 30 min per day. The tasks can be changed according to individual progress.

In addition, a formal contract between the therapist and participant is presented and signed, so that subjects compromise to use their paretic upper limb in activities of daily living as much as possible in the real world, out of the therapeutic setting, as described in constraint-induced movement protocols (39).

### Familiarization

First, subjects are exposed to the interventions in a familiarization session. Either active or sham RPSS is administered, according to the randomization. FES is applied for 5 min.

Measurements of ROM of wrist extension and flexion, grip and pinch strength are performed until subjects reach a stable performance defined as a difference <20% between 3 consecutive measurements. Evaluation of all baseline outcomes is performed on the same day of the familiarization session.

### Outcome Measures

The primary outcome measure is the Wolf Motor Function Test (WMFT) (40) after 6 weeks of treatment (end of treatment). The average time to perform 15 functional tasks (maximum, 120 s per task; if >120 s, the score is 121 s) as well as the quality of movement (Functional Ability Scale; range, 0 to 5) are assessed. The Minimal Clinically Important Differences (MCID) in the chronic phase are 1.2 to 2 s for the average time to complete the tasks, and 0.2 to 0.4 points for the Functional Ability Scale (41). The WMFT is a valid instrument with high interrater and test-retest reliabilities, as well as high internal consistency (42). The original version of the WMFT was developed to assess the effects of CIMT in stroke and traumatic brain injury.

The secondary outcomes are the WMFT at 3, 10 and 18 weeks after beginning of treatment, as well as the following outcomes measured at 3, 6, 10, and 18 weeks after beginning of treatment: MAL; active range of motion (ROM) of wrist extension and flexion; grasp and pinch strength in the paretic and non-paretic sides (the order of testing is randomized within and across subjects); MAS; FMA-UL in the paretic arm; BI; SIS; Beck Depression Inventory Short Form. Measurements assessed after 3 and 6 weeks of treatment are scheduled for days other than those in which the patients receive treatment, in order to avoid fatigue.

In addition, ROM of wrist extension and flexion is measured before and after the first session of treatment. ROM of wrist extension and flexion is measured with an analogic goniometer (ISP® Instituto São Paulo, São Paulo, Brazil) (43). Averages of three trials are calculated. Grip strength is measured with a Jamar dynamometer (Saehan Jamar, Changwong Korea) and pinch strength, with a digital dynamometer (Kratos, São Paulo, Brazil) (44). The trunk and the arms are stabilized and the wrist is kept in a neutral position during measurements.

All outcomes are evaluated by trained researchers blinded to the type of RPSS administered to patients. Assessments are videotaped.

### Adverse Events

Questions about adverse events are asked immediately after each session of RPSS and FES. Subjects are also instructed to spontaneously report any adverse events throughout the protocol.

### Sample Size

We planned to include up to 40 patients and to determine the final sample size after evaluating the results of the first twenty patients included in the trial in regard to the MCID of the primary outcome (change in WMFT, Functional Ability Scale at 6 weeks compared to baseline).

### Data Analysis

Analysis of variance with repeated measures or generalized estimating equations models with factors GROUP (active or sham) and TIME (baseline, 3, 6 weeks) will be performed, according to the distribution of the data. After all follow-ups have been completed, analyses will be performed with factors GROUP (active or sham) and TIME (baseline, 3, 6, 10, and 18 weeks). If there is an imbalance in Fugl-Meyer scores between the groups, then the analysis will be adjusted for baseline severity.

Also, the percentages of patients in the active and sham groups who will attain gains equal or larger than MCID in the WMFT and MAL, FMA-UL at 6, 10, and 18 weeks will be compared with chi-square tests or Fisher's exact tests.

Compliance with the interventions will be compared in the active and sham groups with Mann–Whitney tests.

Intention-to-treat (ITT) and per-protocol analyses will be performed. For intention-to-treat analysis, missing observations will be imputed with the Last Observation Carried Forward (LOCF).

### Data and Safety Monitoring Plan

The P.I. and the research coordinator are responsible for ensuring absolute compliance with eligibility criteria and data confidentiality. All research materials will be kept locked in cabinets. Only the research team members will have a password to use the computer where the data will be acquired and stored. Adverse events are monitored by using Common Toxicity Criteria Manual of the National Cancer Institute as guidance, and reported to the Ethics Committee and to the NIH. Progress reports are sent to the Ethics Committee every 6 months. Serious adverse event reports must be reported to the Ethics Committee within 24 h.

## Discussion

This project represents a major step in developing a rehabilitation strategy with potential to have impact on the treatment of stroke patients with poor motor recovery in developing countries worldwide. The study preliminarily evaluates a straightforward, non-invasive, inexpensive intervention. If preliminary efficacy is demonstrated, further investigations of the proposed intervention (underlying mechanisms/ effects in larger samples of patients) should be performed.

The equipment used for RPSS has yet not received approval for clinical use in Brazil or in the United States. Most studies of RPSS included patients with mild motor impairments. After enrollment of this protocol had started, two studies about effects of RPSS in patients with mild to moderate impairments, conducted by the same group in the United States, were published (11, 45). In one, (45) 19 patients were randomized to 10 sessions of 2-h RPSS followed by 4-h modified constraint-induced therapy for the paretic upper limb, for 10 days. Significantly greater improvements in the WMFT, Action Research Arm Test (ARAT) and FMA-UL were documented after active (*n* = 9), compared to sham stimulation (*n* = 10). The differences in improvements in these outcomes between the groups were also statistically significant 1 month after the end of the intervention. In the other study, (11) 36 patients were included. Similar results were obtained. In addition, improvements in the active group were bigger than the minimal clinically important differences in the Active Research Test. These results were very encouraging, but the feasibility of a 6-h protocol of rehabilitation in the context of developing countries is challenging. Patients with moderate motor impairments often depend on caregivers in order to attend rehabilitation sessions, and the latter may not be able to remain at the health facility where treatment is provided, for a long period of time, over consecutive days. Also, administration of a 4-h modified constraint-induced movement therapy protocol may be a barrier to its implementation in clinical practice in developing and developed countries, considering the costs associated with payment of therapists to deliver treatments in a one-to-one basis over extended periods of time. This difficulty, as well as concerns about fatigue due to long periods of training, fostered development of modified, shorter protocols (46, 47) also shown to be beneficial to subjects in the chronic stage after stroke.

The proposed protocol will thus provide novel information about the effects of RPSS as an add-on intervention to motor training, under a protocol adapted to a developing-world environment, with shorter duration of treatment sessions, distributed over a longer period of time. Furthermore, the protocol has other differences in comparison to the paradigm applied by previous studies: (11, 46) first, recurrent strokes are included in the present protocol but were excluded by others (11, 46). Recurrent strokes are more common in developing countries such as Brazil compared to developed countries, and represented the most common reason for exclusion (45%) in a prior study of neuromodulation in stroke rehabilitation in Brazil (47). Because this protocol intended to be applied in a developing country, recurrent strokes were not excluded.

Second, while Carrico et al. (11) and Yadav et al. (46) administered RPSS to the Erb's point to provide brachial plexus stimulation, in addition to the median and radial nerves, in the current protocol RPSS is administered to the median, radial and ulnar nerves on the wrist/arm as previously reported (33). RPSS with Erb's point stimulation had not been described when the current protocol was designed, while nerve stimulation on the wrist/arm had been previously shown to be well-tolerated (10).

Follow-ups will continue up to 3 months post-treatment in the current protocol, providing information about long-term duration of effects of RPSS as an add-on intervention to treatment. Also, the evaluation of outcomes at 3 weeks aims to investigate whether 9 sessions of treatment lead to similar effects, compared to 18 sessions of treatment. If this hypothesis is proven correct, larger multicenter studies could be conducted faster, and at lower costs. According to current standards of payment of rehabilitation therapy and interventions of non-invasive peripheral stimulation in public health services in Brazil (http://sigtap.datasus.gov.br/tabela-unificada/app/sec/inicio.jsp), the costs of the entire treatment would be approximately 700 Brazilian reals (or 155 dollars).

Finally, detailed imaging data, including DTI analysis, is used in the proposed protocol in order to characterize the degree of involvement of the corticospinal tract, key to motor recovery after stroke, (48) in both the active and sham groups.

In summary, the results of the protocol “Peripheral Nerve Stimulation and Motor Training to Enhance Hand Function after Stroke” will advance the field of non-invasive neuromodulation for upper limb rehabilitation in stroke, a leading cause of disability worldwide.

## Ethics Statement

The protocol, registered at clinicaltrials.gov (identifier, NCT02658578), was approved by the institutional and the Brazilian federal ethics committees (protocol 0546/11, CAAE 70163217.5.0000.0068). The study is being conducted according to the principles of the Declaration of Helsinki (49). Written informed consent is required from all participants or their legal representatives before inclusion.

## Author Contributions

AC and IM wrote the initial draft of the manuscript. AM and LC contributed to study design, revision as well as final approval of the manuscript. NR, RL, DP, CL and EP contributed to revision and final approval of the manuscript.

### Conflict of Interest

The authors declare that the research was conducted in the absence of any commercial or financial relationships that could be construed as a potential conflict of interest.
